# Medical and Philosophical Intelligence

**Published:** 1814-04

**Authors:** 


### MEDICAL AND PHILOSOPHICAL INTELLIGENCE.

ROYAL SOCIETY.—On Thursday the 3d of February, a paper by Sir Humphry Davy was read, entitled *Some further Experiments on Fluorine, and on some other Objects of chemical Investigation.* This paper consisted of three distinct subjects, treated of in succession. 1. Several further experiments were related, made in order to obtain fluorine in a separate state, but they were all unsuccessful. Fluorine has such a tendency to enter into combinations, that no vessels can be procured upon which it does not act. Sir H. Davy considers it as a compound of hydrogen and an unknown supporter of combustion, which he calls *fluorine.* This fluorine has the property of combining with the base of silica and with boron, and it forms an acid with each. The fluates are compounds of fluorine and the metals which constitute the bases of the salifiable bodies. A number of experiments to determine the proportions of the constituents of these bodies were detailed. Fluor spar, when heated with sulphuric acid, increases in weight from 100 to 175·4; but it was requisite to repeat the process eight times to obtain the full effect. If we suppose fluor spar to be a compound of fluoric acid and lime, this result gives us its composition as follows:—

| | |
|---|---|
| Lime . . . . . . | 73·661 |
| Fluoric acid . . . . . . | 26·339 |
| | 100·000 |

But if its constituents are fluorine and calcium, then its composition is—

| | |
|---|---|
| Calcium . . . . . . | 53·313 |
| Fluorine . . . . . . | 46·687 |
| | 100·000 |

According to this statement, an atom of fluorine will weigh 2·294, (supposing an atom of calcium to weigh 2·62).

2. The second part of the paper was on *silica.* A number of unsuccessful attempts to obtain the base of this earth in a separate state were related. They were made by passing potassium through red-hot silica. Potash was obtained mixed with a brown matter, which was converted into silica by the action of water. It would appear that silica contains nearly half its weight of oxygen; and Sir H. Davy conceives it to contain 2 atoms of oxygen to 1 of the base. This would make the weight of an atom of *silicon* (as we may denominate that base) to be 2, nearly or almost the same with that of sulphur.

Sir H. Davy conceives that *silicon* is not a metal, but analogous to *boron* in its nature. These bodies possess intermediate properties between charcoal and sulphur.

3. The third part of the paper was on *chlorine.* A number of unsuccessful attempts to decompose this substance, and obtain oxygen from it, were stated. Sulphuret of lead was fused in it; but not the least trace of sulphate of lead could be obtained. Various experiments of a similar kind were tried, with an equally unsuccessful result. The author noticed the scepticism still entertained by many persons respecting the nature of chlorine, and the arguments brought forward in order to show that it might contain oxygen. Every candid person, he observed, who had seen the combination of dry muriatic acid gas and ammoniacal gas, must be convinced that no more water existed in the compound formed than had previously existed in the gases. If water were really formed, it would indicate rather the decomposition of azote, than the existence of water as a constituent of muriatic acid. The objections of Berzelius from the doctrine of definite proportions are merely apparent, as the one doctrine can be reconciled to these proportions just as easily as the other. The author concluded his paper with some excellent observations on the mode of reasoning in chemistry. Lavoisier had the glory of first introducing sound logic into the science. Chemists may doubt whether there be such a thing as real elements, but they are not at liberty to doubt whether a substance has been decomposed or not, when all attempts to decompound it have failed. Oxygen has been considered as the acidifying principle; but hydrogen forms at least as many acids as oxygen, and it forms several into which oxygen does not enter at all. All cases of combustion were ascribed to the presence and agency of oxygen; but we now know that it takes place whenever bodies combine with energy; and fluorine, chlorine, and iodine, are entitled to the name of supporters, as well as oxygen. He suggests the possibility that the diamond may be a compound of charcoal and some very light unknown supporter.

At the same meeting, a paper, by ANTHONY CARLISLE, Esq. *on Monstrosity in the Human Species,* was read. The author detailed a number of examples of monstrosity, hereditary in particular families, and propagated from one generation to another. All monstrosity he conceives to take place only in cases where the artificial civilization of mankind has interfered. Thus varieties of dogs, pigeons, &c. are easily propagated.

On Thursday the 10th of February, a paper, by A. B. BRODIE, Esq. on *the Influence of the Nerves on the Secretions of the Stomach,* was read. The experiments consisted in cutting the gastric nerves of dogs, and giving them doses of arsenic sufficient to produce death in a few hours. This poison, in common cases, occasions a great secretion of mucus in the stomach and intestines; but in these experiments nothing was found in the stomach after death. Hence the non-secretion of mucus seems owing to the section of the nerves.

At the same meeting a paper was read, by CHARLES KŒNIG, Esq. on the *Human Skeleton from Guadaloupe,* lately deposited in the British tish Museum. Mr. Kœnig introduced his paper by an historical sketch of all the facts that have been ascertained respecting fossil bones. Kamper, Blumenbach, and, above all, Cuvier, are the naturalists that have most distinguished themselves in these researches; but hitherto no human fossil bones had been discovered. Hence it was concluded, either that man was of subsequent creation to those animals, the fossil bones of which have been found, or that if human fossil bones exist, they are covered by the existing ocean, and thus for ever concealed from our sight. The fossil human bones found at Guadaloupe appear to constitute an exception to this general rule. They were discovered by the French governor of Guadaloupe; and the specimen at present in the British Museum was dug up by him at a considerable expence, and was intended for the museum at Paris. The capture of the island by Great Britain enabled Sir Alexander Cochrane to send it to the British Museum.

It was found near the sea shore in a calcareous rock of the hardest texture, being considerably superior in that respect to statuary marble. The rock is partly granular, and partly compact. The granular part is a mixture of grey and flesh-red particles. The red particles Mr. Kœnig considers as the millepora miliacea in fragments; it contains also a few shells. In short, it seems to consist chiefly of a congeries of fragments of coralines connected together firmly without any apparent cement.

The bones of the skeleton are not petrified, but retain the usual constituents of fresh bone; and when first exposed to the air were rather soft. The skull and vertebræ of the neck are wanting. The seven true ribs, and three of the false ribs of the left side remain; but on the right side these bones are destroyed, though the sternal part of the true ribs adhere to those on the left side. The sternum is not visible, being probably sunk into the stone. The dorsal vertebræ are all visible, though not perfectly well defined. The fore-arm and finger bones of one hand remain, and one clavicle. The pelvis is pretty entire, and so are the thigh bones. The legs are so twisted in that the fibula is sunk in the stone.

As to the age of this skeleton, there are no data to form a correct estimate, though in all probability it is not very recent. The appearance of the stone shows decidedly that it does not owe its origin to any calcareous deposition similar to calcareous tuff; but that its formation is analogous to that of common sandstone. It contains traces of phosphate of lime, which seem to demonstrate its animal origin.

On Thursday the 17th of February, a paper, by John Davy, Esq. *on Animal Heat*, was read. The author made a set of experiments in order to determine the specific heat of arterial and venous blood. He employed chiefly the blood of lambs. Two methods of experimenting were followed. 1. The relative times of cooling of equal bulks of arterial and venous blood were determined. The specific gravity of the venous blood was 1·050; that of the arterial 1·047. This method gave the specific heat of arterial blood about 0·93; and that of venous blood 0·92. 2. These two kinds of blood were mixed with

2 x 2 water, water, and the change of temperature marked. The results differed somewhat in different experiments. Arterial blood, by this mode of experimenting, came out of the specific heat 0·95, venous blood 0·94 nearly. It appears to me that these experiments of Mr. Davy are liable to two objections, which must prevent us from putting full confidence in the results which he obtained. 1. It is probable that a chemical action takes place between blood and water; therefore the specific heat of blood cannot be accurately determined by mixing it with water. Suppose we were to mix alcohol and water: the temperature of the mixture would not enable us to determine the specific heat of alcohol. Neither, I am persuaded, would such a mixture enable us to determine the specific heat of blood. 2. Mr. Davy, in his experiments, often drew the venous blood of the animal on one day, and the arterial on another. Now experiments of this kind never lead to accurate results. Whenever you begin to tamper with an animal, you throw it into an unnatural state, and then it is impossible to calculate what sudden changes may be produced on its blood. Mr. Davy made a set of experiments to determine the temperature of arterial and venous blood in animals. The arterial blood was always hottest. In the sheep it was 104° or 105°, while the venous was 103° or 104°. In the ox it was 101°, while the venous was 100°. These results Mr. Davy considers as favourable to Dr. Black's theory of animal heat, and likewise to the notion that the heat depends upon the nervous energy.

---

*Linnæan Society.*—On Tuesday, the 1st of February, a communication from Mr. Reynolds Johnson was read, giving an account of two fossil alcyonia found near Lyme. They were in flint, and in a very perfect state of preservation, and enabled Mr. Johnson to make several additions to our knowledge of the structure of this animal.

At the same meeting a paper was read, by Mr. Roscoe, on the class monandria. It consisted chiefly of strictures on Dr. Roxburgh's essay on that class, in the eleventh volume of the Asiatic Researches.

On Tuesday, February 15, the conclusion of Mr. Roscoe's paper, entitled *Remarks on Dr. Roxburgh's Description of the Monandrous Plants of India*, published in the first volume of the Asiatic Researches, was read. Mr. Roscoe contends for the sufficiency of the generic characters derived from modifications of the anther-bearing filament of Seitamineæ, as proposed by him in his essay on this subject, published in the eighth volume of the Linnæan Transactions; and denies that Dr. Roxburgh's primary characters, taken from differences in the inner limb of the corolla, are of universal importance in this tribe of plants. He then proceeds to remark on certain species described by Dr. Roxburgh, whose *phrynium dichotomum* he regards as probably not specifically different from *marauta arundinacea*. His *phrynium virgatum* he is inclined to refer to *thalia geniculata*. Both Dr. Smith and Dr. Roxburgh have referred *amomum repens* of Sonnerat (the plant producing the lesser cardamoms of the shops) to the the genus alpinia. Mr. Roscoe, however, is induced to agree with Dr. Maton, who, in his observations on Mr. White's paper on cardamoms, published in the tenth volume of the Society's Transactions, has proposed to establish this plant as a new genus, under the name of *elettaria;* and on the authority of the published figures, Mr. Roscoe considers it as differing from alpinia, not only in habit, but in the structure and appendages of its anther-bearing filament.

With regard to Dr. Roxburgh's *globba radicalis,* he adopts the opinion of Dr. Sims, who, in the Botanical Magazine, No. 1428, has established this as a distinct genus, under the name of mantisca saltatoria. Mr. Roscoe considers this genus as differing from globba no less in the structure of its filament than in its inflorescence.

---

*Society for preventing Accidents in Coal Mines.*—A numerous meeting was held at Bishop Wearmouth on the 1st day of October, 1813, Sir Ralph Milbanke, Bart. in the Chair, when it was resolved,—That this meeting, impressed with the frequent and dreadful accidents which have occurred by explosions of inflammable gas, and from other causes, in coal mines, particularly by those which have lately happened in the counties of Durham and Northumberland, notwithstanding the precautionary measures which have been adopted, do form itself into a society, for the purpose of obtaining communications from scientific and practical men as to what modes may be devised for the prevention of such accidents. And a provisional committee was formed for the purpose of forwarding the views of the society.

---

At one of the late meetings of the Westminster Medical Society, a fact was related, which shows the necessity of the practitioner being well acquainted with the relative affinities of different medicinal subtances with each other. A mixture of Minderus's spirit with magnesia was ordered to be given to a patient, but on being administered it produced so alarming a sense of suffocation, that its use could not be continued. The mixture was examined, and found to taste and smell strongly of ammonia. It was now discovered, though never before suspected by the prescriber, that the magnesia possessed a greater affinity for the acetous acid than the ammonia did, and the latter salt was necessarily evolved on the mixture of the two substances.

As this fact is in contradiction to the received tables of affinities published by Dr. Pearson, and as the mistake might be attended with fatal effects in the cases of children, it was important to give this notice of it. It appears, however, on repeating the experiment, that the same effect is not produced by the *carbonate of magnesia,* which we believe is more generally employed in medicine than the other.

---

Amongst the projected institutions of these improving times, we observe a plan for establishing an asylum for lunatics, in or near the metropolis. We hope there is no *cant* about the proposed *humanity* which which is to form a leading principle in the treatment of the patients. If there is really a want of another large receptacle for the forlorn subjects of this disease, every good man will support its establishment; but let not our two great hospitals, and the numerous *private houses*, be insulted by any insinuation that inhumanity or needless harshness is exercised against the unfortunate and helpless inmates of those highly-useful institutions. We know that it is never resorted to in St. Luke's and in Bethlem Hospitals, in which the patients are as comfortable and as happy as the nature of their disease will admit; and with the great competition which now exists among *private houses*, it cannot be supposed that the proprietors, some of whom are medical men of known respectability, would act so directly against their own interests, as to ill-treat the patients committed to their charge.

Another institution we hear is proposed for diseases of the lungs. With great deference for the illustrious and high names that are said to countenance this undertaking, we beg leave to deprecate the measure, as tending to circumscribe practice, and ultimately injure the community. For every guinea subscribed to this charity some other will suffer. Half the dispensaries now existing are supported with difficulty; their means of benefiting the afflicted poor are crippled by a want of funds; and in the face of this, we have a proposal for establishing, at considerable expence, an institution for curing the diseases of one organ only, whereas, in the dispensaries now established, but not adequately maintained, every disease is treated, and no pauper is refused. If it be urged that the man who attends to diseases of the lungs only, will be the most successful in curing them, we deny the assertion. This very minute division of labour so prevalent in our days, tends to narrow the range of intellect, and in the medical profession already approximates to quackery. If a surgeon, for instance, who considered himself an expert cutter of corns and fistulæ, obtained full practice in that useful department of surgery, he very soon would be unfit for any thing else.

---

*New Medical Books published at the Leipzig Michaelmas Fair, in* 1813.

Albrecht, Dr. C. F. sichere Mittel geyen dus Zahnweh. 8vo.

Aemilius Macer et Apuleius A. de Virtutibus Herbarum Recensuit, Dr. G. Seebode. 8vo. Gotting.

Auszuge aus dem Briefwechsel der Gesellschaft correspondirenden Pharmaceuten. Jahrgang, 1813. 8vo.

Beer's Geschichte der Augenkunde uberhaupt und der Augenheilkunde insbesondere. 1s. Heft. gr. 8vo.

——— das Auge, oder Versuch das edelste Geschenk der Schöpfung vor den höchst verderblichlen Einflüssen unseres Zeitalters zu sichern. Mit. Kupf. gr. 8vo.

Beiträge zur Chemie und Physik, in Verbindung mit J. G. Bernhardi, J. Berzelius, &c. Herausgegeben von Dr. J. S. C. Schweiger. 8r. und 9r. Band. gr. 8vo.

Bischoff's Hülfsbüchlein zur Verhütung Vösartigen Fieber. 8vo.

Boer's Versuch einer Darstellung des Kindlichen Organismus. 8vo.

Doctor,

Doctor, der aufrichtige medicinische. 8vo.

Hufeland's guter Rath an Mütter, über die wichtigsten Punkte der rönperlichen Erziehung. 8vo.

——— Geschichte der Gesundheit Eine Skizze. 8vo.

——— und E. Horn's Versuche über die Wirksamkeit des Bernardschen Mittels gegen die Lustseuche. 8vo.

——— und Himly's Journal der Praktischen Heilkunde. Jarhrg. 1812.

——— Bibliothek der praktischen Heilkunde. Jahrg, 1812.

Kopp's Dr. J. H., Jahrbuch den Staatsarzneikunde. 6r. Jahrgang. 8vo.

Michälis, C. F. Abhandlung üben den Steinschnitt u. Verhaltung des Urin's. Mit. Kupf. gr. 4to.

Neuburg's klinische Bemerkungen über einige chronische Krankheiten. 8vo.

Ofterdinger, Dr. G. L. über das Podagra und seine Heilung, nebot Bekauntmachung einer neuen Methode die podagrischen Aufälle zu behaudeln. 8vo.

Octzmann de Fistula Ani. 4to. mag. ann tabula ænea colorata.

Pharmacopœa Borussica. Ed. III. emendata. 8vo. mag.

Pharmacopœa extemporanea Auctore, Dr. F. L. Augustin. Ed. secunda. 8vo.

Ploucquet Litteratura Medica digesta,sive Repertorium Medicinæ practicæ, Chirurgiæ atque rei Obstetricæ. Contumatio et Supplementum Primum.

Reil und Auteurieth's Archie für die Physiologie. 11tes bds. 3tes. Stück. 8vo.

Rheinek's medicinische und chirurgusche Beobachtungen über die einfache Methode des Seiten-Blasen-Steinschnitts. 8vo.

Richter, A. G. die Specielle Therapie, nach den hinterlassenen Papieren des Verstorbenen. Herausg. von Dr. G. A. Richter. 1r. bd. Die Acuten Krankheiten. 8vo.

——— Medicinische und Chirurgische Bemerkungen, aus einem hinterlassenen Manuscripte herausg. 2r. bd. 8vo.

Siebold Chiron, cine der theor. prakt. u. historischen Bearbeitung der Chirurgie gewidmete Zeitschrift. 3 ten. bds. 2 tes Stück. 8vo.

Spangenberg, Disquisitio inaug. anat. circa partes genitales fœmineas Avium c. tab. V. æn. 4to.

Stein's Annalen der Geburtshülfe. 4 ter. Janog. 8vo.

Teuffel's Magazin für theoretische und praktische Thierheilkunde. 1 ter. bd.

Trawnitschek über die Natur und Heilung des Nasencatarrhs. 8vo.

Tromensdorff's Journal der Pharmacie. 22n. bds. 2 tes. Stück.

Wurzeri D. F. Analysis Chemica Calculi renalis equini. 4to.

——— Narratio de Analysi Urinæ insolitæ. 4to.

Zeitung Medicinisch-chirurgische, Fortgesetzt von Dr. J. N. Ehsart. Jahrg. 1813.

*Preparing for Publication.*

Auteurieth's, J. H. F. Von, Handbuch der empirischen meuschlichen Physiologie, 3 bde Zweite Aufl. 8vo.

Macquer's

Macquer's Chymisches Wüterbuchneu bearbeitet von Dr. Sigism. Friedr. Hermbstädt. 4ten. bd. 8vo.

Sammlung, neue, auserleseuen Wahrnchmungen aus dem Gebiete der praktischen Medizin u. Chirurgie. 1 ster. Theil. gr. 8vo.

Woerterbuch, medicinisches, oder Realencyclopædie der Arzueikunst in Alphabetischer Ordnung. 8vo.

---

An Oration has been instituted, by the liberality of Dr. Baillie and Sir Everard Home, Bart. (who have each contributed 500*l.*) in commemoration of the late John Hunter. This oration is to be delivered *in perpetuo* on the anniversary of the natal day of that celebrated surgeon, (Feb. 14), at the Royal College of Surgeons.

The first oration was delivered on Monday the 14th instant, before a most crouded auditory; among whom were Earl Spencer, the President of the Royal Society, Sir Archibald Macdonald, Right Hon. George Rose, Sir Francis Milman, Dr. Baillie, Sir Henry Halford, and many other distinguished members of the sister College, public teachers, &c. Sir Everard Home, Master of the College of Surgeons, was the orator.

Sir E. commenced by detailing the objects for which the oration was instituted. He conceived himself highly honored that it should have devolved on him, while master, to deliver the first oration, an office in which the two governors, Sir William Blizard and Mr. Cline, were so eminently qualified to have preceded, but which they both had joined in conferring on him. He proceeded to notice the just distinction that had been made between medicine and surgery; and professed his conviction of the propriety of such distinction being preserved, as the only true means that would advance each to that degree of perfection of which it was capable. He then entered on a review of the merits of those surgeons who had rendered their names most celebrated since the erection of the corporation.

To Wiseman we were chiefly indebted as the first who, by his works, gave a degree of importance to surgery which before it never possessed, and thus made the first step to raise it from an art and mystery, which it was before considered, to the rank of a science. Before his time, surgeons were mechanics. By his talents, and his conduct, he elevated the character of the surgeon, and emancipated him from the wheels of the physician's chariot, to which he was before enchained.

Cheselden's genius and superior attainments, which early in life acquired him fame and the highest honors of his profession, were forcibly commented on. To him we were obliged for an admirable System of Anatomy, which the author lived to see pass through six editions; and for a valuable work on Osteology. He became renowned above all his contemporaries by a new and more successful method of operating for the stone; while his penetration admirably elucidated the physiology of vision, by his philosophical observations on the well-known case of the youth on whom he successfully operated for congenital cataract. Cheselden's Theory of Vision the orator illustrated, by remarks deduced from his own experience.

Sir Cæsar Hawkins he mentioned, not from any work that he had left of the results of his practice, but because he was the inventor of the cutting gorget for the operation of the stone, an instrument that had been universally approved and adopted, as diminishing the danger of the operation, and which, except in very young or lean subjects, could not be so well performed by any other yet discovered.

Of the abilities, observations, and improvements, introduced into the art of surgery by Pott, whose labours were continued through half of the last century, ample notice was taken. His works on Hernia, on Injuries of the Head, and on Fractures of the Limbs, were proofs of his talents and his industry. His reasoning on Fistula in Ano—his adoption of a simple operation for one most painful and often hazardous, by the mere division of the rectum, and his invention of the curved bistory for performing it, evinced the soundness of his judgment, the fertility of his resources, and his expertness as an operator.

Contemporary with Pott, though thirty years junior in practice, was John Hunter, who became a student of St. Bartholomew's Hospital in 1748. In a few years he removed to St. George's, where he pursued his studies with all that ardour and assiduity that, even in his later years, so strongly marked a perfect devotion to his profession. During this period he assisted his brother, Dr. William Hunter, and contributed, by his ingenuity and indefatigable labours, to form that Museum which was left by the doctor to the University of Glasgow. About 1760 he received an appointment as staff surgeon in the army. In 1763 he was elected surgeon to St. George's Hospital; and in 1766 first commenced his surgical lectures.

His acuteness, his perseverance, his physiological experiments, his unbounded desire for research, which, not contented with the study of the human body, impelled him to extend his inquiries by comparative anatomy to the brute creation, were cursorily noticed.

The orator then gave a succinct account of the discoveries of Mr. Hunter; neatly eulogising his character, talents, acquirements, and habits; and justly holding up his example as highly worthy of imitation by the junior part of his auditory. "He was employed from the rising to the setting of the sun, and frequently continued the greater part of the night in his favourite studies; and in making those invaluable anatomical preparations that formed the museum which, by the munificence of the legislature, now adorns this College."

Sir E. apologised for confining himself to this brief sketch of a man of whom so much might be said; but he was aware that others would succeed him in that chair, by whom that which he had omitted would be well supplied. It was not his intention, he said, to draw comparisons. At this epoch there existed so many members of that College, eminent for their abilities as anatomists and surgeons, and for their devotion to science, that an attempt to assign pre-eminence where so many excelled were vain. He congratulated himself that it was his good fortune to live in times when the art of surgery had advanced its claims to the highest consideration, and its professors to be classed among the sons of science. Yet, just as those claims were, he acknowledged that the science of medicine ought to rank the first in the healing art; and that among the members of the Royal College of Physicians were men of the greatest learning and erudition, possessing all the varied qualifications essential to the superior branch of the profession.

The orator's peroration was dignified and impressive.

---

Mr. W. Henley, Pharmaceutical Chemist, has ascertained that barilla does not contain any of the new principle denominated *iodine*; and that the quantity to be obtained from kelp is very variable, sometimes it depositing itself with the crystallised salts of the evaporated solution, at other times it remaining in the uncrystallizible residuum. From his experiments he has every reason to conclude that it is a compound of two well-known substances; and the results of his investigation he hopes very shortly to be enabled to lay before the scientific world.

---

A correspondent informs us that he has successfully punctured the membrane of the tympanum in three cases of deafness, with complete success, and without any relapse of the complaint. The method employed was the improved operation recommended by Mr. Cellies.

A trocar was used three or four times the usual size, the cutting angles of which were also made larger and sharper, that the chance of the re-union of the incised membrane might be lessened; and the opening was made at the inferior and anterior part, to avoid the handle of the malleus. This situation appeared more favourable for the puncture, on account of the vessels and nerves; for although the wounding of them is not ordinarily attended with inconvenience, it seemed possible that a sufficient hæmorrhage *might* take place to furnish a coagulum capable of blocking up the cavity of the tympanum, which would render the operation abortive.

---

We are requested to state, that in a pamphlet circulated by Dr. Walker's Vaccine Institution, the name of Mr. Lawrence, of the College of Physicians, has been inserted, without his consent or authority, as Assistant-Director of that establishment. Mr. L. has never had, directly or indirectly, any share whatever in directing the affairs of that institution; nor has he had any other connection whatever with it, than that of contributing a small annual subscription to its funds.

---

Dr. Robert Watt, of Glasgow, will begin his summer course of Lectures on the Practice of Medicine, on Tuesday the 3d of May next, at nine in the morning.

---

According to the report of several *Apothecaries* residing in various districts of the metropolis, the following was the average of the diseases in London between January 20th and February 19th:—

| | | | |
|---|---|---|---|
| Anasarca . . . | 15 | Amenorrhœa . . | 22 |
| Ascites . . . | 19 | Asthenia . . | 56 |
| Asthma . . | 126 | Abortio . . . | 23 |
| Acne . . . | 8 | Bronchitis acuta . . | 16 |
| Apoplexia . . | 21 | ——— chronica . | 30 |
| Aphtha lactantium . . | 13 | Cancer . . | 4 |

| | |
|---|---|
| Chlorosis | 14 |
| Cholera | 16 |
| Chorea | 3 |
| Cephalalgia | 55 |
| Colica | 39 |
| Catarrhus | 347 |
| Convulsio | 20 |
| Cynanche Tonsillaris | 79 |
| ——— maligna | 12 |
| ——— Trachealis | 7 |
| ——— parotidea | 14 |
| Diabetes | 2 |
| Dysenteria | 30 |
| Diarrhœa | 126 |
| Dyspnœa | 44 |
| Dyspepsia | 95 |
| Dysuria | 20 |
| Ecthyma | 4 |
| Eczema | 6 |
| Erythema papulatum | 7 |
| Erysipelas | 38 |
| Enteritis | 9 |
| Enterodynia | 7 |
| Enuresis | 3 |
| Epilepsia | 5 |
| Febris remittens | 86 |
| —— intermittens | 23 |
| —— puerpera | 7 |
| Gastrodynia | 33 |
| Hæmatemesis | 2 |
| Hæmoptoe | 24 |
| Hæmorrhois | 24 |
| Hepatitis | 28 |
| Hysteria | 26 |
| Hysteritis | 5 |
| Hydrocephalus | 16 |
| Hydrothorax | 11 |
| Herpes | 31 |
| Hypochondriasis | 9 |
| Icterus | 14 |
| Icthyosis | 1 |
| Impetigo | 6 |
| Ischuria | 6 |
| Leucorrhœa | 28 |
| Lithiathis | 2 |
| Lepra | 4 |
| Lichen | 2 |
| Mania | 15 |
| Menorrhagia | 36 |
| Miliaria | 3 |
| Morbi Infantiles | 140 |
| ——— Biliosi | 111 |
| Nephritis | 7 |
| Obstipatio | 46 |
| Ophthalmia | 59 |
| Paralysis | 22 |
| Phthisis Pulmonalis | 40 |
| Pleurodyne | 10 |
| Pertussis | 78 |
| Peritonitis | 5 |
| Phrenitis | 7 |
| Pneumonia | 145 |
| Podagra | 27 |
| Porrigo larvalis | 10 |
| ——— scutulata | 6 |
| ——— favosa | 4 |
| Psoriasis | 21 |
| Purpura | 2 |
| Pyrosis | 4 |
| Rachitis | 1 |
| Rheumatismus acutus | 85 |
| ——— chronicus | 124 |
| Rubeola | 18 |
| Scabies | 72 |
| Scarlatina simplex | 20 |
| ——— anginosa | 7 |
| ——— maligna. | 4 |
| Scrofula | 17 |
| Synochus | 5 |
| Tabes Mesenterica | 5 |
| Typhus | 6 |
| Variola | 11 |
| Varicella | 12 |
| Vermes | 34 |
| Vertigo | 25 |
| Urticaria | 12 |
| Total | 3046 |

The Deaths in the Bills of Mortality from January 18th to February 15th, 1814, according to the returns of the *Parish Clerks*, were as under:—

| | |
|---|---|
| Males | 967 |
| Females | 944 |
| Total | 1911 |

